# Comprehensive geriatric assessment for people living with HIV and frailty: A mixed‐methods feasibility randomized controlled trial

**DOI:** 10.1111/hiv.70149

**Published:** 2025-11-21

**Authors:** Natalie St Clair‐Sullivan, Katherine Bristowe, Stephen Bremner, Matthew Maddocks, Richard Harding, Thomas Levett, Jonathan Roberts, Zoe Adler, Peter May, Gary Pargeter, Jaime H. Vera

**Affiliations:** ^1^ Cicely Saunders Institute of Palliative Care, Policy & Rehabilitation King's College London London UK; ^2^ Florence Nightingale Faculty of Nursing, Midwifery & Palliative Care, King's College London London UK; ^3^ Department of Global Health and Infection Brighton and Sussex Medical School Brighton UK; ^4^ University Hospitals Sussex NHS Trust Brighton UK; ^5^ School of Medicine Trinity College Dublin Dublin Ireland; ^6^ Lunch Positive Brighton UK

**Keywords:** comprehensive geriatric assessment, feasibility studies, frailty, HIV, human immunodeficiency virus, outpatient

## Abstract

**Objective:**

Prevalence of geriatric syndromes including frailty among people living with HIV is increasing and at younger ages. There is no gold standard model of care for people with HIV and frailty. This study aimed to determine the acceptability of a comprehensive geriatric assessment and management plan, delivered jointly by a geriatrician and HIV physician (the Silver Clinic) in outpatient HIV services, and also the feasibility of conducting a randomized controlled trial (RCT) of the Silver Clinic compared with standard care.

**Design:**

Mixed‐methods single‐centre, parallel, two‐arm feasibility RCT.

**Methods:**

People living with HIV ≥50 years old, who screened as frail using the FRAIL scale were randomized to: usual care or the Silver Clinic. Randomization was stratified by age and sex, target *N* = 84. The primary objective was to determine whether a definitive trial is feasible.

**Results:**

Twenty‐five participants (46% of *n* = 55 eligible patients) were randomized. One hundred percent of participants attended their 6‐month follow‐up and 91% at 12 months. More than 90% of the outcome measures were completed at all time points. Interviews revealed study processes and outcome measures were acceptable, and that the intervention was valued by people living with HIV and frailty.

**Conclusions:**

Delivering a comprehensive geriatric assessment jointly by a geriatrician and HIV physician was feasible and acceptable. Retention and completion of outcome measures were high, although recruiting sufficient frail individuals from one site was challenging. A RCT to determine the effectiveness of the Silver Clinic is warranted, but will require a multicentre design and an extended recruitment period to address recruitment challenges.

## INTRODUCTION

Ageing people living with HIV face a higher burden of comorbidities, social care challenges and geriatric conditions, such as frailty and cognitive decline, compared with the general population [1]. These significantly impact mental well‐being, functional capabilities and quality of life, and increase healthcare utilization and associated costs [2].

There is no gold standard model of care for the management of people living with HIV and frailty [3]. While there is growing recognition of the need to address geriatric syndromes within HIV care settings [4], existing HIV care provision lacks the necessary expertise or resources to manage geriatric syndromes effectively [5]. The European AIDS Clinical Society now recommends routine frailty screening for people living with HIV aged 50 and above, followed by a comprehensive geriatric assessment for those identified as frail [6]. The comprehensive geriatric assessment, the gold standard for managing frailty in older people, is a multidimensional assessment of an older person, including health and well‐being, to create an individualized care plan [7]. However, published data from geriatric clinics for people living with HIV are limited, and there is little evidence to support the efficacy of frailty screening, or the role of the comprehensive geriatric assessment in improving health outcomes for people living with HIV [5, 8].

This study aimed to determine the acceptability of a comprehensive geriatric assessment and frailty management plan, delivered jointly by a geriatrician and HIV physician (the Silver Clinic) in outpatient HIV services, and the feasibility of conducting an randomized controlled trial (RCT) of the Silver Clinic compared with standard care. Specifically, it examined the feasibility of trial procedures and participant retention, initial cost and service utilization analysis and areas for refinement in referral pathways, clinic frameworks and intervention strategies.

## METHODS

### Study design

A UK‐based mixed‐methods single‐centre, parallel, two‐arm feasibility RCT among older people living with HIV (≥50 years old), screened as frail using the FRAIL scale. Participants were randomized to either the intervention group (comprehensive geriatric assessment and management plan, delivered jointly by a geriatrician and HIV physician in outpatient HIV services, known as the ‘Silver Clinic’) or standard care. A full description of the protocol is available elsewhere [9].

### Setting

Participants were recruited from the HIV unit at the Royal Sussex County Hospital, Brighton, UK.

### Recruitment

All potentially eligible patients who attended their routine HIV annual health visit during the study recruitment period were identified and screened. This visit is a standard procedure, as part of usual HIV care in the United Kingdom. Performed by nurses, it encompasses weight assessment, blood pressure monitoring, urinalysis, mental health evaluation, sexual health screening, adherence assessment, cervical cytology and contraception review. During this assessment, individuals aged 50 and above underwent frailty screening utilizing the FRAIL scale [10]. 1572/2457 (64%) attending the Lawson Unit are 50 years and over and 60 (4%) are women [11]. The FRAIL scale was selected due to it being recommended by the European AIDS Clinical Society, its ease of use, and that it does not have space implications within the clinic [11]. Telephone‐based frailty screening was also conducted with participants from a previous study who had consented to be contacted, and patients that were identified as pre‐frail the previous year were rescreened. Those exhibiting signs of frailty on screening were informed about the study and, if interested, connected with a research assistant or nurse to provide further information, and address any queries. Written informed consent was obtained before any data collection commenced at their baseline visit.

### Inclusion criteria

- People living with HIV aged 50 years or older with evidence of frailty, scoring 3+ on the FRAIL scale.
- Able to provide informed consent.
- Consented to their general practitioner being informed about participation.

### Exclusion criteria

- People living with HIV aged <50 years old or not defined as frail.
- Attended the Silver Clinic previously during the last 12 months.

### Data collection

Data were collected simultaneously in both groups at baseline (T1), 26 weeks (T2, primary end point) and 52 weeks (T3) using standardized clinical outcome measures. Due to challenges in recruitment, the initial recruitment period was extended by 2 months. Due to time restrictions, those recruited after September 2022 had only T1 and T2 follow‐ups. The Montreal Cognitive Assessment (MoCA) was initially planned to be conducted at T1 only. Subsequent ethical approval was obtained to include the MoCA at T2 and T3 after the trial had commenced.

### Patient demographic and characterization data

Baseline demographic data were collected including personal characteristics (age, gender, sex at birth, ethnicity, sexual orientation) and social determinants of health (marital status, employment status, residential status, formal education level, annual income) and comorbidities.

### Feasibility outcomes

#### Primary outcome

To determine whether a definitive trial is feasible, assessed by:

- Recruitment rate
- Study retention
- Completion of study outcome measures

#### Secondary outcomes

##### Frailty and patient‐reported outcome measures

Secondary outcomes were captured at all time points (T1, T2 and T3) via standardized clinical outcome measures that represent multiple health and healthcare service domains. The Timed Up and Go test (TUGT) is used to assess functional mobility and falls risk [12]; the Adult Social Care Outcomes Toolkit (ASCOT) measures social care‐related quality of life [13]; the EuroQol EQ‐5D­5L measures health‐related quality of life [14]; the Positive Outcomes HIV PROM (HIV PROM) measures multidimensional symptoms and concerns for people living with HIV [15]; the MoCA assesses early detection of mild cognitive impairment [16]; the Consultation and relational empathy measure (CARE) is used to assess interpersonal quality of healthcare encounters [17]; the FRAIL scale and the Fried Frailty Phenotype [18] are used to assess physical frailty.

##### Health service use

The Client Service Receipt Inventory (CSRI) [19] captures planned and unplanned contact with general practitioners, hospital and emergency department attendance and admissions, and informal care by family and/or friends, to measure the costs of caring for older people living with HIV and frailty.

### Description of interventions

#### Intervention arm: The Silver Clinic

The Silver Clinic is a pre‐existing, joint HIV–ageing outpatient service that was first established in 2012 to address the emerging needs of older adults living with HIV. The Silver Clinic consists of a comprehensive geriatric assessment and management plan, delivered jointly by a geriatrician and HIV physician in outpatient HIV services at the Royal Sussex County Hospital. The clinic uses a comprehensive geriatric assessment approach that involves the comprehensive evaluation and management of geriatric syndromes commonly observed in older people living with HIV, such as frailty, falls, polypharmacy, multimorbidity and medication‐related issues associated with antiretroviral therapy (ART) [9]. Additionally, the intervention provides support for older people living with HIV facing social and psychological challenges by devising patient‐centred health interventions including physical activity promotion and peer support. Each clinic appointment involves a structured process comprising history‐taking, physical examination, collection of blood samples, medication review, cognitive assessment and evaluation of social and mental health aspects. An individualized care plan is formulated and communicated to the patient's primary care provider or HIV physician for coordination of care. All participants had a separate general practitioner.

The clinic operates on a monthly basis offering individual appointments of 40 min to up to 16 patients per month. Follow‐up requirements are determined by the Silver Clinic physicians, in line with the needs of the patient. Silver Clinic appointments are offered both in‐person and virtually to ensure ease of access to the service.

#### Control arm: Standard care

**FIGURE 1:**
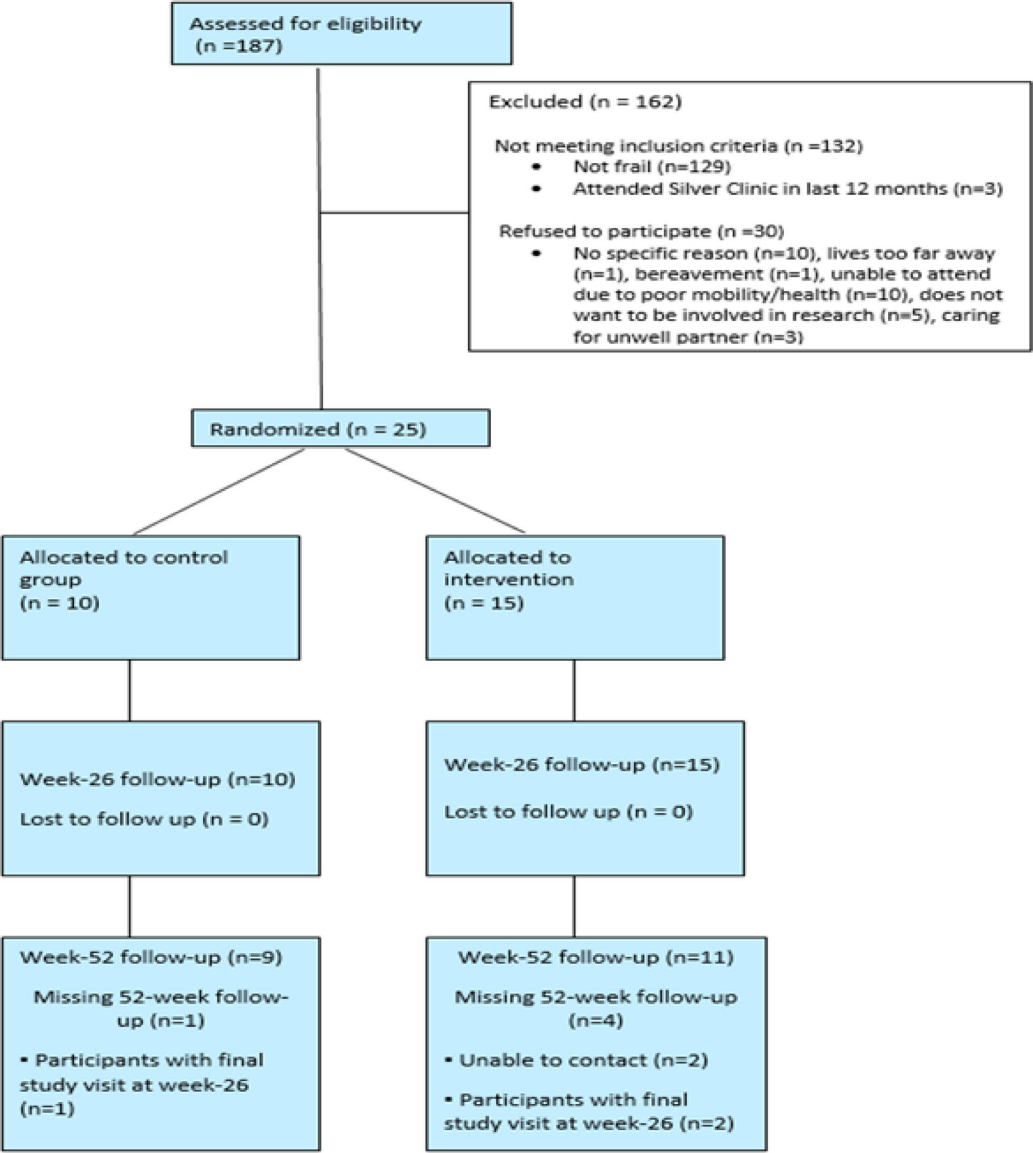
CONSORT flow diagram.

Participants randomized to the control arm continued to receive routine healthcare services from their HIV physician, general practitioner and community resources as per standard practice (see Figure 1). All participants had a separate general practitioner. HIV standard care is typically provided twice a year, with primary care services predominantly accessed upon patient request. Participants were provided with information regarding frailty and consented to the disclosure of their frailty assessment outcomes to their GP and HIV physician. They received general healthy ageing advice but did not have access to the intervention during the study. Upon completion of the 12‐month study period, control group participants were offered an opportunity to attend the Silver Clinic.

### Randomization and blinding

After baseline assessment, the research assistant randomized participants using REDCap in a 1:1 allocation using randomly permuted blocks to receive standard care (control arm) or referral to the Silver Clinic (intervention arm), stratifying by age (50–65, 66–80, 81–95) and sex at birth to ensure a balanced sample in both arms. Participants were informed of their study arm in person and those allocated to the intervention arm were booked into the Silver Clinic within 6 weeks.

#### A priori progression criteria to a definitive trial

To guide decisions on trial feasibility, we established the following a priori progression criteria. Meeting these thresholds indicated the study could progress to a definitive trial without substantive changes.

- Recruitment of 60% of eligible patients;
- Recruitment of 84 patients within 3 months; from first patient randomized;
- Retention of 70 patients (allowing up to 15% attrition) to primary end point (6 months); and
- Outcome measure completion for 90% of available participants at each time point.

### Sample size

To accurately estimate the standard deviation of the primary outcome for the subsequent trial, it is recommended to have a minimum of 35 participants per arm [20, 21]. Considering our local patient population (total cohort of 2450; 54% aged over 50), we planned to recruit 42 patients per group, totalling 84 participants overall, with a 15% allowance for attrition, allowing us to estimate retention to within approximately ±8% with 95% confidence.

### Statistical analysis

Baseline characteristics of the participants were summarized using descriptive statistics. Normally distributed variables were summarized by their means and SD, skewed continuous variables by their medians and interquartile ranges and categorical variables by their frequencies and percentages. For the feasibility outcomes, the proportion of patients recruited, participants retained and data completeness were analysed and summarized in line with the a priori progression criteria and 95% CIs are presented.

### Explanatory qualitative interviews

We aimed to interview up to 15 participants from each arm of the trial, via purposive sampling to examine experiences of: recruitment to the trial, management of their priority concerns during the course of the trial, referral to the Silver Clinic, the description of comprehensive geriatric assessment, experience of the Silver Clinic and perceived impact on priority outcomes (intervention arm only), satisfaction with care and acceptability of participating in an RCT of the Silver Clinic intervention. Due to recruitment challenges for the main trial, a decision was made to invite all trial participants to participate in a qualitative interview in order to ensure sufficient information power to address the aim of the study [22].

### Qualitative analysis

Interviews were audio‐recorded, transcribed verbatim and analysed using reflexive thematic analysis, using the six‐phase process [23, 24]. Commencing with immersion in the data the second phase involved generating initial codes, which are refined through an iterative process into themes and sub‐themes in the subsequent phases, to accurately depict the participants' experiences [25]. Coding was led by a physiotherapist (NS) with experience in qualitative research and overseen by the qualitative lead for the study (KB). The themes and findings were reviewed and refined with the study team.

### Patient and public involvement

Patient and public involvement was embedded through the development and conduct of the study, including recruitment strategies and participant‐facing materials, and dissemination of study findings.

### Ethical approval

Ethical approval was granted by East Midlands–Leicester Central Research Ethics Committee (reference 21/EM/0200). The study was registered at www.isrctn.com (ISRCTN14646435).

## RESULTS

### Recruitment and retention

Recruitment took place between November 2021 and March 2023. The last participant visit was completed in September 2023. Of *n* = 187 individuals assessed for eligibility, *n* = 162 (86.7%) were not eligible (*n* = 129 did not screen as frail, *n* = 3 had attended the Silver Clinic in the last 12 months). Of the *n* = 55 who were eligible, *n* = 30 (54.5%) declined to take part (*n* = 10 gave no specific reason, *n* = 1 lived too far away, *n* = 1 had experienced a bereavement, *n* = 10 were unable to due to poor health/mobility, *n* = 5 did not want to be involved in research and *n* = 3 were caring for an unwell partner). *N* = 25 (45.5%, 95% CI = 32.0, 59.4) were recruited and randomly allocated to intervention (*n* = 15) or usual care (*n* = 10) (Figure 1). One hundred percent of participants attended their T2 follow‐up (primary endpoint) and 91.0% at T3.

### Participant characteristics

Most participants were male (99%) and White (88%), with a median (IQR) age of 64 (54–79) years. Participants had been living with HIV for a median (IQR) of 24 (16.5, 34) years, on ART for a median (IQR) of 19 (9, 25) years, a median (IQR) of 6 (5, 9) comorbidities. See Table 1 for baseline characteristics by randomized group.

**TABLE 1:** Descriptive statistics (frequency and percentage unless otherwise indicated) of baseline characteristics by trial arm and overall.

	Intervention *N* = 15	Control *N* = 10	All *N* = 25
Age (years)^a^	62.6 (5.95)	65.1 (6.2)	63.6 (6.1)
Time on ART (years)^a^	18 [8, 23]	23 [10.3, 26.8]	19 [9, 25]
On ART *n* (%)			
Yes	15 (100)	9 (90)	24 (96)
No	0 (0)	1 (10)	1 (4)
Viral Load <40 copies	15 (100)	10 (100)	25 (100)
Last CD4 count^b^	795 [557, 966]	715.5 [525, 1134.3]	759 [550, 1006]
Time since diagnosis (years)^b^	24 [14, 25]	33 [18.2, 37]	24 [16.5, 34]
Fall last 6 months (number)^b^	1 [0, 6]	0 [0,2]	0 [0,4.5]
Number of non‐ART medications^b^	9 [5.0, 10]	10 [5.6, 10]	10 [5.5, 10]
FRAIL scale score	3 (3–4)	3 (3–4)	3 (3–4)
Sexual orientation			
Heterosexual or straight	1 (6.7)	0 (0)	1 (4)
Gay or lesbian	11 (73.3)	9 (90)	20 (80)
Bisexual	1 (6.7)	1 (10)	2 (8)
Other	2 (13.3)	0 (0)	2 (8)
Ethnicity			
White British	11 (73.3)	9 (90)	20 (80)
Black African	1 (6.7)	0 (0)	1 (4)
Black & White Caribbean	2 (13.3)	0 (0)	2 (8)
White other	1 (6.7)	1 (10)	2 (8)
Charlson Comorbidity Index^a^	2.7 (1.3)	3.2 (1.2)	2.9 (1.3)
Number of comorbidities^a^	6 [5, 9]	5 [5, 9]	6 [5, 9]
Sex			
Male	14 (93.3)	10 (100)	24 (96)
Female	1 (6.7)	0 (0)	1 (4)
Gender			
Male	14 (93.3)	9 (90)	23 (92)
Female	1 (6.7)	0 (0)	1 (4)
Gender fluid	0 (0)	1 (10)	1 (4)
Marital status			
Single (never married)	7 (46.7)	7 (70)	14 (56)
Married/domestic partnership	4 (26.7)	1 (10)	5 (20)
Widowed	1 (6.7)	1 (10)	2 (8)
Divorced	2 (13.3)	1 (10)	3 (12)
Separated	1 (6.7)	0 (0)	1 (4)
Education	0 (0)	2 (20)	2 (8)
Primary school	3 (20)	3 (30)	6 (24)
Secondary school up to 16 years Higher/further education	3 (20)	2 (20)	5 (20)
College or university	6 (40)	2 (20)	8 (32)
Post‐graduate degree	2 (13.3)	1 (10)	3 (12)
Prefer not to say	1 (6.7)	0 (0)	1 (4)
Household annual income			
Below £10 000	2 (13.3)	1 (10)	3 (12)
£10 001–£20 000	6 (40)	6 (60)	12 (48)
£20 001–£30 000	1 (6.7)	2 (20)	3 (12)
£30 001–£40 000	0 (0)	0 (0)	0 (0)
Above £40 000	4 (26.7)	0 (0)	4 (16)
Prefer not to say	2 (13.3)	1 (10)	3 (12)
Present home			
Owner occupied/owned outright	4 (26.7)	1 (10)	5 (20)
Owner occupied/ mortgage	1 (6.7)	1 (10)	2 (8)
Rented housing/council assoc.	9 (60)	5 (50)	14 (56)
Rented/private landlord	1 (6.7)	3 (30)	4 (16)
Employment status			
Employed, 1–39 h/pw	0 (0)	1 (10)	1 (4)
Employed, 40+ h/pw	0 (0)	0 (0)	0 (0)
Not employed, not looking	1 (6.7)	1 (10)	2 (8)
Retired	8 (53.3)	3 (30)	11 (44)
Disabled, not able to work	5 (33.3)	5 (50)	10 (40)
Prefer not to say	1 (6.7)	0 (0)	1 (4)

^a^ Mean (SD).

^b^ Median [IQR].

### Completion rates for outcome measures

99.7% of outcome measures were completed at baseline, 99.0% at T2 and 90.2% at T3. Data were missing in the TUGT (4% at T2, 17.2% at T3), due to a participant not being able to physically walk that distance, a participant who felt too frail to attend their T2 and T3 visit in person and two participants not attending their T3 visit (reason unknown). As the follow‐up MoCAs were introduced as an amendment to the study, two MoCAs (8%) were not completed at T2, due to those participants already having had their T2 visit and two MoCAs (9.1%) were not completed at T3 due to participants not attending.

### Outcome measure results

Overall, satisfaction with care was high in all areas of the CARE measure, with ≥70% rating their practitioner as excellent, very good or good at T2 and T3 visits (see Appendix A). Improvements were seen in the intervention arm in the TUGT (T1 = 15, T3 = 12), ASCOT (T1 = 0.6, T3 = 0.71) and the EQ5D5L VAS (T1 = 50, T3 = 57), at T3 rather than T2 (Table 2). The intervention arm also reported fewer hospital days, GP contacts, consultant contacts and Allied health professional contacts between T1 and T3.

**TABLE 2:** Descriptive statistics of frailty and patient‐reported outcomes by trial arm and time point.

	Intervention			Control		
	T1 (*n* = 15)	T2 (*n* = 15)	T3 (*n* = 11)	T1 (*n* = 10)	T2 (*n* = 10)	T3 (*n* = 9)
TUGT^	15 [11, 18]	15 [12, 20]	12 [10, 16]	12 [6.8, 20]	12 [10, 22]	13 [10, 23]
SCRQoL (ASCOT)+	0.6 [0.4, 0.74]	0.5 [0.4, 0.9]	0.71 [0.4, 0.9]	0.6 [0.33, 0.74]	0.6 [0.41, 0.7]	0.5 [0.4, 0.8]
EQ5D5L VAS+	50 [50, 65]	50 [40, 65]	57 [50, 75]	58 [39, 65]	65 [44, 79]	50 [41, 60]
HIV PROM+	30.7 (7.1)	28.7 (10.0)	28.3 (9)	29.0 (9.3)	24.8 (8.4)	25.5 (12.2)
MOCA+	26 [24, 27]	25 [23, 26.3]	26 [24, 27]	26.5 [23, 28.3]	24 [21, 27]	25 [24, 28]
FRAIL scale^	3 [3, 3]	3 [3, 3]	3 [2, 3]	3 [3, 3]	3 [2, 3.3]	2 [2, 3]
FRIED^	3 [3, 3]	3 [3, 3]	3 [3, 3]	3 [2, 3]	3 [2, 3]	3 [2, 3]
Hospital days	0 (0)	1 (4)	0 (0)	0 (0)	2.4 (7)	1.2 (4)
GP contacts	4 (3.1)	1.5 (2)	3 (4)	1 (1)	1 (2)	8.1 (20.3)
Consultant contacts	1.8 (1.2)	2 (1.6)	1.5 (1.4)	1 (1)	1.3 (1.1)	1.7 (1.3)
AHP contacts	2.3 (7.4)	1.1 (3.1)	1.1 (3)	0.1 (0.3)	0.1 (0.3)	2.4 (7)

*Note*: Mean (SD), Median [IQR]; T1: baseline, T2: 6 months, T3: 12 months. Missing data – Timed Up and Go test: T1 *n* = 1 intervention, T2 *n* = 1 intervention, T3 *n* = 4 intervention; Montreal Cognitive Assessment: T2 *n* = 1 intervention, *n* = 1 control, T3 *n* = 2 intervention; all other measures: T3 *n* = 2. +higher scores better, ^higher score worse. Contacts refer to patient appointments or interactions with the named healthcare professional (consultant, AHP or GP). Allied health professionals (AHP) include: physiotherapists, occupational therapists, dieticians, podiatrists, speech and language therapists, radiographers. Consultant contacts: Hospital consultant physicians (senior doctors in the UK healthcare system).

### Trial qualitative interview findings

All but one participant from the main trial completed qualitative interviews (telephone *n* = 17, video call *n* = 1, HIV research unit *n* = 6), after their T2 study visit. The sample consisted of 22 men, one woman and one person who identified as gender fluid. Interviews lasted up to 1 h (range 35–60 min).

Interviews revealed that participants found the trial processes and communication to be acceptable and clear. Participants largely described a feeling of neutral acceptance towards the outcome measures and questionnaires used during the trial, often describing them as *‘fine’* and *‘par for the course’*. However, participants discussed how they may identify issues that they themselves may not have been aware of. Overall participants described their experiences of attending the Silver Clinic as positive, particularly in relation to their mental well‐being. Suggestions on how to improve the Silver Clinic were centred around improving linkage of care and increasing follow‐up appointments (e.g., quotes see Table 3).

**TABLE 3:** Participant quotes.

Experiences of participating in the trial	*‘You were very specific about you know, either you'll be, as I've just said, in four visits or, you know, you'll need to go to the Silver Clinic and review you that way, yeah, so yeah, very clear.’* (P04, intervention arm) *‘Actually I had a bit of fun. We had a few laughs, it helped the time go by.’* (P07, control arm) *‘I didn't feel it felt rushed, and you know, it was a good use of time, it wasn't like you were just sitting around there waiting. No, I felt an hour was… yeah, it was perfectly acceptable level of time. You didn't feel like you were wasting time, and you didn't feel you were being rushed.’* (P19, intervention arm) ‘*You've made me feel very safe, you know, when you are talking to me… You are not a person that will make me feel threatened or harassed or anything, even the doctor that I saw also really made me feel at home. Yeah, thank you so much for having me in the study.’* (P17, intervention arm) *‘It makes me feel good…It's like giving something back to all these people, you know, to the HIV and the research department.’* (P25, control arm)
Feelings around outcomes measures	*‘I can understand the reason for them, and see the logic behind them, I don't have a problem with them.’* (P14, control arm) ‘*Okay, some of them are a bit obscure, and whatever but they're just… you answer the question as it is, try and not to think too much about it, and then eventually the information that's required will be collated and put together and then come out. So, yeah, I mean I… that to me is fine*.’ (P11, intervention arm) *‘There are the needs that I identify as my needs towards better health, but then there are… there are needs that a professional person would identify as what I need for better health, and you know, the two aren't necessarily compatible, and to be quite honest, I think you know people often think that “if I had this, I would be so much healthier” or “I would be so much better off” and very often…a lot more objective perspective from ascertaining what the problems are can actually give a better… can give a better idea of what the needs are, identifying the issues, the problems.’* (P10, intervention arm)
Experiences of the Silver Clinic	*‘If it wasn't for the Silver Clinic, I wouldn't have had the physiotherapy for my knee and that wouldn't have made me question about the problems that I'm having with my shoulder by going to the doctor and… I would have just lived with it. But because I had the good experience of like, you know, getting physiotherapy done and stuff, okay, right I can… there are people that are willing to listen, it made me think, you know, “Yeah, you've got a problem, how can you fix it?”’* (P11, intervention arm) *‘I'm feeling a million times better, because I know., that I'm not suffering from heart disease … which has hugely reduced my stress levels. So, it was great, it was just all so, you know, it was all so… it was holistic, it was, you know, you guys have treated me as a person, not just as somebody with HIV.’* (P19, intervention arm) *‘There were a number of people there that were professionals and you can vent your own emotional problems that you're having at the time. You can explain to them like “I'm in pain constantly” … You're discussing it and you're getting your emotions out I suppose. A bit of therapy.’* (P03, intervention arm) *‘It's made my mind settled a lot, and it's made me feel… yeah, it's given me a new lease of life.’* (P22, intervention arm)
Ways to improve the Silver Clinic	*‘The only thing I would say is chasing the results was a bit of a nightmare… It reverted back to the sort of the old you* versus *the GP.’* (P19, intervention arm) *‘I can't think of the word, but sort of try and, you know, bring it all under one umbrella and so there would be, you know, better dialogue between departments and all this sort of thing.’* (P02, intervention arm) *‘It's having the ability to actually follow things through on a one‐to‐one basis… Actually go back to the Lawson Unit and say “Well, this hasn't been done, this hasn't been done, and this has got to be sorted out now.”’* (P13, intervention arm)

## DISCUSSION

### Key findings

This study aimed to determine the acceptability of a comprehensive geriatric assessment and management plan, delivered jointly by a geriatrician and HIV physician (the Silver Clinic) in outpatient HIV services, and the feasibility of conducting an RCT of the Silver Clinic compared with standard care. We found that due to recruitment challenges, conducting a full‐scale RCT using the current methodology would not be feasible. However, all other trial progression criteria were met, including high participant retention (100% at 6 months and 91% at 12 months) and high rates of outcome measure completion (99.7% at baseline, 99% at 6 months, 90.2% at 12 months). Our qualitative findings further support the acceptability of the study processes and outcome measures, with participants expressing positive experiences, including enjoyment of their study visits. Those in the intervention arm uniformly reported that attending the Silver Clinic was beneficial and considered it a valuable addition to their HIV care. Participants also provided useful feedback on potential improvements to the intervention, including direct access to test results, improved coordination of care and more frequent follow‐ups. Consistent with previous research, these findings underscore the interconnected nature of physical and mental health and highlight the potential for physical health interventions to confer secondary benefits on mental well‐being [26, 27]. The potential for the term frailty to be perceived as stigmatizing is an important issue within both people living with HIV and care provider communities. As we have explored these issues in depth elsewhere [28, 29], the present paper focuses on feasibility outcomes. This study also demonstrated that screening people living with HIV aged ≥50 years using the FRAIL scale was both feasible and acceptable to participants. Identification of frailty is particularly critical, as frailty has been shown to predict poor health outcomes and is negatively associated with adherence to ART [30]. While multiple frailty screening tools exist, there is no consensus on which is a ‘gold standard’ tool [31] and is reliable and simple enough for routine clinical settings [32]. Additionally, there are no screening tools that are validated in people living with HIV [33] and many that are validated are in populations that are 65 years or over [34]. Evidence suggests that the Fried Frailty Phenotype is not suited for implementation in large‐scale population studies or busy clinics and that short, rapid instruments are more appropriate [31]. It is also important to recognize that the FRAIL scale is a screening tool, whereas the diagnosis of frailty typically relies on the Frailty Phenotype or other established diagnostic metrics. Analysis of secondary outcomes suggests that frailty screening and management pathways may contribute to improved health outcomes over the longer term, with benefits observed at 12 rather than 6 months. This highlights the importance of extending trial durations when evaluating interventions for conditions such as frailty, where improvements in physical function and adaptation to new management strategies may take longer to manifest [35, 36]. While these findings should be interpreted with caution due to the small sample size and feasibility focus of the study, they provide a foundation for future hypothesis‐driven research and underscore the necessity of conducting a definitive RCT of a comprehensive geriatric assessment for older people living with HIV with frailty.

Although evidence for the use of the comprehensive geriatric assessment in pre‐frail populations is limited, including among people living with HIV, pre‐frailty is increasingly recognized as a ‘warning sign’ for impending frailty [31] and therefore represents an important window for identification and timely intervention to reduce the risk of further deterioration. As pre‐frail individuals are at increased risk of becoming frail within 3–4 years [37], early recognition and intervention are likely to provide the greatest benefit [38, 39]. In people living with HIV, early identification of frailty could enable access to interventions that may delay or reverse progression, thereby reducing disability, hospitalization and mortality [40]. Future work should examine how the comprehensive geriatric assessment might be optimally delivered in this context and its potential role in addressing pre‐frailty.

To improve the feasibility of a future trial, we propose expanding recruitment criteria to include pre‐frail individuals, who comprise an estimated 25–30% of people living with HIV aged ≥50 years [41]. Screening for and intervening in pre‐frailty offers an opportunity to slow or reverse frailty progression, particularly among individuals in their 50s who may be more receptive to interventions aimed at maintaining long‐term health. A shift from a reactive to a proactive model of care may therefore be more appropriate and acceptable [42]. Additionally, recruitment is expected to improve as clinical services resume face‐to‐face appointments, particularly for individuals with complex healthcare needs. Expanding the number of trial sites offering a comprehensive geriatric assessment will also enhance recruitment and generalizability.

### Strengths and limitations

This study has several limitations. Participants were not blinded to their group allocation, although HIV physicians and geriatricians conducting the Silver Clinic were unaware of who the trial participants were. The sample predominantly comprised White males, reflecting the ongoing issue of underrepresentation of women in HIV clinical trials [43]. Future trials should prioritize inclusive recruitment strategies, such as extending research sites to diverse geographical locations, engaging women's health specialists and collaborating with women's advocacy groups to build trust and encourage participation [44].

Recruitment fell short of expectations due to delays associated with the COVID‐19 pandemic, including staff redeployment, hospital service disruptions and patient reluctance to attend in‐person visits. To mitigate these challenges, we extended the recruitment period (resulting in some participants receiving only 6 months of follow‐up), recruited patients who had participated in previous studies, rescreened individuals previously identified as pre‐frail, and implemented telephone‐based frailty screening. These adaptations demonstrated the feasibility and acceptability of administering the FRAIL scale remotely, offering a time‐efficient alternative with minimal implications for clinic space.

Missing outcome data were primarily attributed to participant mobility limitations, which affected their ability to attend study visits or complete assessments, an issue commonly observed in frailty‐related research [45]. This highlights the importance of offering flexible means to participation (in person or virtually) for a future trial. While missing data in frailty measures may serve as an indicator of frailty severity, it could also result in misclassification, with participants being categorized as pre‐frail rather than frail due to an inability to complete certain measures. Furthermore, while the FRAIL scale demonstrated feasibility and acceptability, further validation against other screening tools is warranted to ensure optimal accuracy and reliability.

Despite these limitations, this study provides valuable new insights. High participant satisfaction with both trial procedures and the Silver Clinic intervention reinforces the acceptability of the study design. Additionally, the successful use of telephone‐based FRAIL screening highlights the potential for integrating remote screening methods into future frailty‐related trials. Key considerations for a definitive RCT include optimizing recruitment strategies, offering flexible participation options (virtual and in‐person), refining screening methodologies and minimizing missing data during assessments.

## CONCLUSION

While recruitment to a full‐scale RCT is not feasible using the current methodology, retention rates and study outcome completion were exceptionally high. Future trials should adopt a proactive recruitment strategy by expanding the number of study sites, potentially expanding to include pre‐frail individuals, and incorporating flexible participation options. These adaptations may enhance feasibility and ensure broader applicability. A definitive RCT remains essential to evaluate the effectiveness of the comprehensive geriatric assessment in improving health outcomes for older people living with HIV with frailty. If successful, this work could inform the integration of comprehensive geriatric assessment within routine HIV outpatient care, ultimately supporting healthy ageing and improved quality of life in this population.

## AUTHOR CONTRIBUTIONS

JV was the grant award holder and chief investigator, and KB was the principal investigator and provided project oversight. JV, KB, NS, MM, RH, SB, TL and GP contributed to the study design. NS collected the data and data analysis was led by NS, KB, JV and SB. NS drafted the first version of the manuscript. All authors contributed to the interpretation of the data revised the manuscript and gave final approval of the manuscript.

## FUNDING INFORMATION

This project is funded by the National Institute for Health and Care Research (NIHR) under its Research for Patient Benefit (RfPB) Programme (Grant Reference Number NIHR201060). The views expressed are those of the author(s) and not necessarily those of the NIHR or the Department of Health and Social Care.

## CONFLICT OF INTEREST STATEMENT

The authors have no conflicts of interest to declare.

## Data Availability

The data that support the findings of this study are available on request from the corresponding author. The data are not publicly available due to privacy or ethical restrictions.

## APPENDIX A

CARE outcome measure (*n*, %)

**Table:**

CARE	Intervention			Control		
	Visit 1 (*N* = 15)	Visit 2 (*N* = 15)	Visit 3 (*N* = 11)	Visit 1 (*N* = 10)	Visit 2 (*N* = 10)	Visit 3 (*N* = 9)
How good was your practitioner at making you feel at ease?						
Excellent	11 (73.3)	11 (73.3)	10 (90.9)	6 (60)	8 (80)	5 (55.6)
Very good	4 (26.7)	3 (20)	1 (9.1)	1 (10)	1 (10)	3 (33.3)
Good	0	1 (6.7)	0	2 (20)	1 (10)	1 (11.1)
Fair	0	0	0	1 (10)	0	0
How good was your practitioner at letting you tell your ‘story’?						
Excellent	8 (53.3)	10 (66.7)	8 (72.7)	5 (50)	7 (70)	4 (44.4)
Very good	6 (40)	4 (26.7)	3 (27.3)	2 (20)	0	4 (44.4)
Good	1 (6.7)	1 (6.7)	0	2 (20)	3 (30)	1 (11.1)
Does not apply	0	0	0	1 (10)	0	0
How good was your practitioner at really listening?						
Excellent	11 (73.3)	10 (66.7)	7 (63.6)	5 (50)	8 (80)	4 (44.4)
Very good	4 (26.7)	3 (20)	4 (36.4)	3 (30)	0	5 (55.6)
Good	0	2 (13.3)	0	2 (20)	1 (10)	0
Fair	0	0	0	0	1 (10)	0
How good was your practitioner at being interested in you as a whole person?						
Excellent	11 (73.3)	8 (53.3)	7 (63.6)	6 (60)	6 (60)	5 (55.6)
Very good	1 (6.7)	4 (26.7)	4 (36.4)	1 (10)	1 (10)	3 (33.3)
Good	3 (20)	2 (13.3)	0	3 (30)	3 (30)	1 (11.1)
Does not apply	0	1 (6.7)	0	0	0	0
How good was your practitioner at fully understanding your concerns?						
Excellent	8 (53.3)	7 (46.7)	7 (63.6)	5 (50)	7 (70)	4 (44.4)
Very good	3 (20)	6 (40)	4 (36.4)	2 (20)	0	4 (44.4)
Good	2 (13.3)	2 (13.3)	0	3 (30)	3 (30)	1 (11.1)
Fair	1 (6.7)	0	0	0	0	0
Does not apply	1 (6.7)	0	0	0	0	0
How good was your practitioner at showing care and compassion?						
Excellent	9 (60)	9 (60)	8 (72.7)	5 (50)	7 (70)	5 (55.6)
Very good	4 (26.7)	5 (33.3)	3 (27.3)	3 (30)	0	3 (33.3)
Good	2 (13.3)	1 (6.7)	0	1 (10)	3 (30)	1 (11.1)
Poor	0	0	0	1 (10)	0	0
How good was your practitioner at being positive?						
Excellent	10 (66.7)	7 (46.7)	7 (63.6)	6 (60)	6 (60)	4 (44.4)
Very good	2 (13.3)	6 (40)	3 (27.3)	1 (10)	3 (30)	4 (44.4)
Good	2 (13.3)	1 (6.7)	1 (9.1)	3 (30)	1 (10)	1 (11.1)
Fair	0	1 (6.7)	0	0	0	0
Does not apply	1 (6.7)	0	0	0	0	0
How good was your practitioner at explaining things clearly?						
Excellent	10 (66.7)	9 (60)	8 (72.7)	6 (60)	7 (70)	4 (44.4)
Very good	3 (20)	3 (20)	3 (27.3)	1 (10)	1 (10)	3 (33.3)
Good	1 (6.7)	2 (13.3)	0	1 (10)	2 (20)	2 (22.2)
Fair	1 (6.7)	1 (6.7)	0	1 (10)	0	0
Does not apply	0	0	0	1 (10)	0	0
How good was your practitioner at helping you to take control?						
Excellent	8 (53.3)	7 (46.7)	7 (63.6)	4 (40)	5 (50)	4 (44.4)
Very good	2 (13.3)	5 (33.3)	1 (9.1)	3 (30)	0	3 (33.3)
Good	0	2 (13.3)	1 (9.1)	1 (10)	4 (40)	2 (22.2)
Fair	2 (13.3)	1 (6.7)	2 (18.2)	1 (10)	0	0
Poor	0	0	0	1 (10)	0	0
Does not apply	3 (20)	0	0	0	1 (10)	0
How good was your practitioner at making a plan of action with you?						
Excellent	7 (46.7)	7 (46.7)	6 (54.4)	3 (30)	5 (50)	3 (33.3)
Very good	0	4 (26.7)	0	3 (30)	0	3 (33.3)
Good	1 (6.7)	2 (13.3)	1 (9.1)	1 (10)	2 (20)	3 (33.3)
Fair	2 (13.3)	1 (6.7)	1 (9.1)	1 (10)	1 (10)	0
Poor	1 (6.7)	1 (6.7)	1 (9.1)	1 (10)	0	0
Does not apply	4 (26.7)	0	2 (18.2)	1 (10)	2 (20)	0

CONSORT 2010 checklist of information to include when reporting a pilot or feasibility trial*.

**Table:**

Section/Topic	Item No	Checklist item	Reported on page No
**Title and abstract**			
	1a	Identification as a pilot or feasibility randomised trial in the title	1
	1b	Structured summary of pilot trial design, methods, results, and conclusions (for specific guidance see CONSORT abstract extension for pilot trials)	2
**Introduction**			
Background and objectives	2a	Scientific background and explanation of rationale for future definitive trial, and reasons for randomised pilot trial	4
	2b	Specific objectives or research questions for pilot trial	4
**Methods**			
Trial design	3a	Description of pilot trial design (such as parallel, factorial) including allocation ratio	5
	3b	Important changes to methods after pilot trial commencement (such as eligibility criteria), with reasons	6
Participants	4a	Eligibility criteria for participants	6
	4b	Settings and locations where the data were collected	5
	4c	How participants were identified and consented	5
Interventions	5	The interventions for each group with sufficient details to allow replication, including how and when they were actually administered	7/8
Outcomes	6a	Completely defined prespecified assessments or measurements to address each pilot trial objective specified in 2b, including how and when they were assessed	8/9
	6b	Any changes to pilot trial assessments or measurements after the pilot trial commenced, with reasons	6
	6c	If applicable, prespecified criteria used to judge whether, or how, to proceed with future definitive trial	8/9
Sample size	7a	Rationale for numbers in the pilot trial	9
	7b	When applicable, explanation of any interim analyses and stopping guidelines	n/a
Randomization:			
Sequence generation	8a	Method used to generate the random allocation sequence	8
	8b	Type of randomisation(s); details of any restriction (such as blocking and block size)	8
Allocation concealment mechanism	9	Mechanism used to implement the random allocation sequence (such as sequentially numbered containers), describing any steps taken to conceal the sequence until interventions were assigned	8
Implementation	10	Who generated the random allocation sequence, who enrolled participants, and who assigned participants to interventions	8
Blinding	11a	If done, who was blinded after assignment to interventions (for example, participants, care providers, those assessing outcomes) and how	8/22
	11b	If relevant, description of the similarity of interventions	
Statistical methods	12	Methods used to address each pilot trial objective whether qualitative or quantitative	9/10
**Results**			
Participant flow (a diagram is strongly recommended)	13a	For each group, the numbers of participants who were approached and/or assessed for eligibility, randomly assigned, received intended treatment, and were assessed for each objective	11
	13b	For each group, losses and exclusions after randomisation, together with reasons	10/11
Recruitment	14a	Dates defining the periods of recruitment and follow‐up	10
	14b	Why the pilot trial ended or was stopped	
Baseline data	15	A table showing baseline demographic and clinical characteristics for each group	12
Numbers analysed	16	For each objective, number of participants (denominator) included in each analysis. If relevant, these numbers should be by randomised group	15/16
Outcomes and estimation	17	For each objective, results including expressions of uncertainty (such as 95% confidence interval) for any estimates. If relevant, these results should be by randomised group	15/16
Ancillary analyses	18	Results of any other analyses performed that could be used to inform the future definitive trial	17–20
Harms	19	All important harms or unintended effects in each group (for specific guidance see CONSORT for harms)	
	19a	If relevant, other important unintended consequences	
**Discussion**			
Limitations	20	Pilot trial limitations, addressing sources of potential bias and remaining uncertainty about feasibility	22
Generalizability	21	Generalizability (applicability) of pilot trial methods and findings to future definitive trial and other studies	21/22
Interpretation	22	Interpretation consistent with pilot trial objectives and findings, balancing potential benefits and harms, and considering other relevant evidence	21/22
	22a	Implications for progression from pilot to future definitive trial, including any proposed amendments	22
**Other information**			
Registration	23	Registration number for pilot trial and name of trial registry	10
Protocol	24	Where the pilot trial protocol can be accessed, if available	5
Funding	25	Sources of funding and other support (such as supply of drugs), role of funders	1
	26	Ethical approval or approval by research review committee, confirmed with reference number	10

*Note*: Citation: Eldridge SM, Chan CL, Campbell MJ, Bond CM, Hopewell S, Thabane L, et al. CONSORT 2010 statement: extension to randomised pilot and feasibility trials. BMJ. 2016;355. This is an Open Access article distributed in accordance with the terms of the Creative Commons Attribution (CC BY 3.0) license (http://creativecommons.org/licenses/by/3.0/), which permits others to distribute, remix, adapt and build upon this work, for commercial use, provided the original work is properly cited. *We strongly recommend reading this statement in conjunction with the CONSORT 2010, extension to randomised pilot and feasibility trials, Explanation and Elaboration for important clarifications on all the items. If relevant, we also recommend reading CONSORT extensions for cluster randomised trials, non‐inferiority and equivalence trials, non‐pharmacological treatments, herbal interventions, and pragmatic trials. Additional extensions are forthcoming: for those and for up‐to‐date references relevant to this checklist, see www.consort-statement.org.

## References

[1] Sarma P , Cassidy R , Corlett S , Katusiime B . Ageing with HIV: medicine optimisation challenges and support needs for older people living with HIV: a systematic review. Drugs Aging. 2023;40:179‐240. 36670321 10.1007/s40266-022-01003-3 PMC9857901

[2] Greene M , Shi Y , Boscardin J , Sudore R , Gandhi M , Covinsky K . Geriatric conditions and healthcare utilisation in older adults living with HIV. Age Ageing. 2022;51(5):1‐9. 10.1093/ageing/afac093 PMC9271234 35511728

[3] Davis AJ , Greene M , Siegler E , et al. Strengths and challenges of various models of geriatric consultation for older adults living with human immunodeficiency virus. Clin Infect Dis. 2022;74(6):1101. 34358303 10.1093/cid/ciab682 PMC8946774

[4] Kehler DS , Milic J , Guaraldi G , Fulop T , Falutz J . Frailty in older people living with HIV: current status and clinical management. BMC Geriatr. 2022;22(1):919. 36447144 10.1186/s12877-022-03477-7 PMC9708514

[5] Kokorelias KM , Grosse A , Zhabokritsky A , Sirisegaram L . Understanding geriatric models of care for older adults living with HIV: a scoping review and qualitative analysis. BMC Geriatr. 2023;23(1):417. 37422631 10.1186/s12877-023-04114-7 PMC10329351

[6] European AIDS Clinical Society . EACS Guidelines Version 11.0. 2021 https://www.eacsociety.org/media/final2021eacsguidelinesv11.0_oct2021.pdf

[7] Garrard JW , Cox NJ , Dodds RM , Roberts HC , Sayer AA . Comprehensive geriatric assessment in primary care: a systematic review. Aging Clin Exp Res. 2020;32:197‐205. 30968287 10.1007/s40520-019-01183-w PMC7033083

[8] Jones HT , Levett T , Barber TJ . Frailty in people living with HIV: an update. Curr Opin Infect Dis. 2022;35(1):21‐30. 34799510 10.1097/QCO.0000000000000798

[9] St Clair‐Sullivan N , Bristowe K , Adler Z , et al. Silver Clinic: protocol for a feasibility randomised controlled trial of comprehensive geriatric assessment for people living with HIV and frailty. BMJ Open. 2023;13(5):e070590. 10.1136/bmjopen-2022-070590 PMC10201220 37208140

[10] Abellan Van Kan G , Rolland Y , Bergman H , Morley JE , Kritchevsky SB , Vellas B . The I.A.N.A. task force on frailty assessment of older people in clinical practice. J Nutr Health Aging. 2008;12(1):29‐37.18165842 10.1007/BF02982161

[11] St Clair‐Sullivan N , Bristowe K , Khan I , et al. Implementation of frailty screening for older people living with HIV in Brighton, UK. HIV Med. 2023;25:1‐7. 10.1111/hiv.13598 38062917

[12] Richardson S . The timed “up & go”: a test of basic functional mobility for frail elderly persons. J Am Geriatr Soc. 1991;39(2):142‐148. 1991946 10.1111/j.1532-5415.1991.tb01616.x

[13] Netten A , Burge P , Malley J , et al. Outcomes of social care for adults: developing a preference‐weighted measure. Health Technol Assess (Rockv). 2012;16(16):1‐165. 10.3310/hta16160 22459668

[14] Rabin R , de Charro F . EQ‐5D: a measure of health status from the EuroQol group. Ann Med. 2001;33:337‐343. 11491192 10.3109/07853890109002087

[15] Bristowe K , Murtagh FEM , Clift P , et al. The development and cognitive testing of the positive outcomes HIV PROM: a brief novel patient‐reported outcome measure for adults living with HIV. Health Qual Life Outcomes. 2020;18(1):1‐10. 32631444 10.1186/s12955-020-01462-5 PMC7336444

[16] Nasreddine Z , Phillips N , Bédirian V , et al. The Montreal cognitive assessment, MoCA: a brief screening tool for mild cognitive impairment. J Am Geriatr Soc. 2005;53:695‐699. 15817019 10.1111/j.1532-5415.2005.53221.x

[17] Mercer SW , Maxwell M , Heaney D , Watt GCM . The consultation and relational empathy (CARE) measure: development and preliminary validation and reliability of an empathy‐based consultation process measure. Fam Pract. 2004;21(6):699‐705. 15528286 10.1093/fampra/cmh621

[18] Fried LP , Tangen CM , Walston J , et al. Frailty in older adults: evidence for a phenotype. J Gerontol A Biol Sci Med Sci. 2001;56(3):M146‐M157. doi:10.1093/gerona/56.3.M146 11253156

[19] Beecham J , Snell T , Perkins M , Knapp M . Health and social care costs for young adults with epilepsy in the UK. Health Soc Care Community. 2010;18(5):465‐473. 20491967 10.1111/j.1365-2524.2010.00919.x

[20] Sim J , Lewis M . The size of a pilot study for a clinical trial should be calculated in relation to considerations of precision and efficiency. J Clin Epidemiol. 2012;65(3):301‐308. 22169081 10.1016/j.jclinepi.2011.07.011

[21] Teare MD , Dimairo M , Shephard N , Hayman A , Whitehead A , Walters SJ . Sample size requirements to estimate key design parameters from external pilot randomised controlled trials: a simulation study. Trials. 2014;15(1):1‐13. 24993581 10.1186/1745-6215-15-264 PMC4227298

[22] Malterud K , Siersma VD , Guassora AD . Sample size in qualitative interview studies: guided by information power. Qual Health Res. 2016;26(13):1753‐1760. 26613970 10.1177/1049732315617444

[23] Braun V , Clarke V . Reflecting on reflexive thematic analysis. Qual Res Sport, Exerc Health. 2019;11(4):589‐597.

[24] Braun V . In: Clarke V , ed. Successful Qualitative Research: a Practical Guide for Beginners. SAGE; 2013.

[25] Braun V , Clarke V . Supporting best practice in reflexive thematic analysis reporting in palliative medicine: a review of published research and introduction to the reflexive thematic analysis reporting guidelines (RTARG). Palliat Med. 2024;38:608‐616. doi:10.1177/02692163241234800 38469804 PMC11157981

[26] Pearce M , Garcia L , Abbas A , et al. Association between physical activity and risk of depression: a systematic review and meta‐analysis. JAMA Psychiatr. 2022;79:550‐559. 10.1001/jamapsychiatry.2022.0609 PMC9008579 35416941

[27] Marquez DX , Aguinãga S , Vásquez PM , et al. A systematic review of physical activity and quality of life and well‐being. Transl Behav Med. 2020;10(5):1098. 33044541 10.1093/tbm/ibz198 PMC7752999

[28] St Clair‐Sullivan N , Vera JH , Maddocks M , et al. ‘We are fragile, but we are strong’: A qualitative study of perspectives, experiences and priority outcomes for people living with HIV and frailty. HIV Med. 2024;26:1‐11. 10.1111/hiv.13722 39400445

[29] St Clair‐Sullivan N , Simmons K , Harding‐Swale R , et al. Frailty and frailty screening: a qualitative study to elicit perspectives of people living with HIV and their healthcare professionals. HIV Med. 2022;24:1‐11. 10.1111/hiv.13419 36229192

[30] Iriarte E , Cianelli R , de Santis J . Frailty in the context of older people living with HIV: a concept analysis. Adv Nurs Sci. 2021;44. 10.1097/ANS.0000000000000384 34718255

[31] Deng Y , Sato N . Global frailty screening tools: review and application of frailty screening tools from 2001 to 2023. Intractable Rare Dis Res. 2024;13(1):1. 38404737 10.5582/irdr.2023.01113 PMC10883846

[32] Buta BJ , Walston JD , Godino JG , et al. Frailty assessment instruments: systematic characterization of the uses and contexts of highly‐cited instruments. Ageing Res Rev. 2016;26:53‐61. 26674984 10.1016/j.arr.2015.12.003 PMC4806795

[33] Beanland A , Alagaratnam J , Goffe C , et al. Objective and subjective rapid frailty screening tools in people with HIV. HIV Med. 2021;22(2):146‐150. 33151034 10.1111/hiv.12988

[34] Pelloquin R , Abdo M , Mawhinney S , Jankowski CM , Erlandson KM . Physical function and frailty tools in mortality prediction of middle‐aged adults with HIV. J Acquir Immune Defic Syndr. 2020;85(3):372‐378. 32732769 10.1097/QAI.0000000000002455 PMC7572796

[35] Mandolesi L , Polverino A , Montuori S , et al. Effects of physical exercise on cognitive functioning and wellbeing: biological and psychological benefits. Front Psychol. 2018;9:509. 29755380 10.3389/fpsyg.2018.00509 PMC5934999

[36] Araújo‐Soares V , Hankonen N , Presseau J , Rodrigues A , Sniehotta FF . Developing behavior change interventions for self‐Management in Chronic Illness: an integrative overview. Eur Psychol. 2019;24(1):7. 31496632 10.1027/1016-9040/a000330 PMC6727632

[37] Krankowska DC , Załęski A , Wiercińska‐Drapało A . Frailty and prefrailty in people living with HIV, with focus on women living with HIV. Int J STD AIDS. 2022;33:1106‐1110. 36217985 10.1177/09564624221127744

[38] Erlandson KM , Piggott DA . Frailty and HIV: moving from characterization to intervention. Curr HIV/AIDS Rep. 2021;18(3):157‐175. 33817767 10.1007/s11904-021-00554-1 PMC8193917

[39] Sacha J , Sacha M , Sobon J , Borysiuk Z , Feusette P . Is it time to begin a public campaign concerning frailty and pre‐frailty? A review article. Front Physiol. 2017;8:484. 28744225 10.3389/fphys.2017.00484 PMC5504234

[40] Liu S , Yan Q , Jiang Y , et al. The impact of frailty on all‐cause mortality in patients with HIV infection: a systematic review and meta‐analysis. AIDS Res Hum Retrovir. 2022;38(9):692‐699. 35658605 10.1089/AID.2021.0155

[41] Yamada Y , Kobayashi T , Condo A , et al. Prevalence of frailty and prefrailty in people with human immunodeficiency virus aged 50 or older: a systematic review and meta‐analysis. Open Forum Infect Dis. 2022;9(5):1‐10. 10.1093/ofid/ofac129 PMC8995074 35415198

[42] Lauretani F , Longobucco Y , Ferrari Pellegrini F , et al. Comprehensive model for physical and cognitive frailty: current organization and unmet needs. Front Psychol. 2020;11:569629. 33324282 10.3389/fpsyg.2020.569629 PMC7725681

[43] Johnston CD , O'Brien R , Côté HCF . Inclusion of women in HIV research and clinical trials. AIDS. 2023;37(6):995. 37017020 10.1097/QAD.0000000000003514 PMC10081809

[44] Barr E , Marshall LJ , Collins LF , et al. Centring the health of women across the HIV research continuum. Lancet HIV. 2024;11(3):e186‐e194. 38417977 10.1016/S2352-3018(24)00004-3 PMC11301651

[45] Boreskie KF , Hay JL , Boreskie PE , Arora RC , Duhamel TA . Frailty‐aware care: giving value to frailty assessment across different healthcare settings. BMC Geriatr. 2022;22(1):13. 34979966 10.1186/s12877-021-02722-9 PMC8722007

